# Dietary Protein Quantity, Quality, and Exercise Are Key to Healthy Living: A Muscle-Centric Perspective Across the Lifespan

**DOI:** 10.3389/fnut.2019.00083

**Published:** 2019-06-06

**Authors:** Nicholas A. Burd, Colleen F. McKenna, Amadeo F. Salvador, Kevin J.M. Paulussen, Daniel R. Moore

**Affiliations:** ^1^Department of Kinesiology and Community Health, University of Illinois, Urbana, IL, United States; ^2^Division of Nutritional Sciences, University of Illinois, Urbana, IL, United States; ^3^Faculty of Kinesiology and Physical Education, University of Toronto, Toronto, ON, Canada

**Keywords:** children, adolescents, aging-old age-seniors, skeletal muscle mass, muscle protein synthesis/breakdown, leucine, anabolic

## Abstract

A healthy eating pattern, regardless of age, should consist of ingesting high quality protein preferably in adequate amounts across all meals throughout the day. Of particular relevance to overall health is the growth, development, and maintenance of skeletal muscle tissue. Skeletal muscle not only contributes to physical strength and performance, but also contributes to efficient macronutrient utilization and storage. Achieving an optimal amount of muscle mass begins early in life with transitions to “steady-state” maintenance as an adult, and then safeguarding against ultimate decline of muscle mass with age, all of which are influenced by physical activity and dietary (e.g., protein) factors. Current protein recommendations, as defined by recommended dietary allowances (RDA) for the US population or the population reference intakes (PRI) in Europe, are set to cover basic needs; however, it is thought that a higher protein intake might be necessary for optimizing muscle mass, especially for adults and individuals with an active lifestyle. It is necessary to balance the accurate assessment of protein quality (e.g., digestible indispensable amino acid score; DIAAS) with methods that provide a physiological correlate (e.g., established measures of protein synthesis, substrate oxidation, lean mass retention, or accrual, etc.) in order to accurately define protein requirements for these physiological outcomes. Moreover, current recommendations need to shift from single nutrient guidelines to whole food based guidelines in order to practically acknowledge food matrix interactions and other required nutrients for potentially optimizing the health effects of food. The aim of this paper is to discuss protein quality and amount that should be consumed with consideration to the presence of non-protein constituents within a food matrix and potential interactions with physical activity to maximize muscle mass throughout life.

## Introduction

The development of a healthy eating pattern, or the identification of the best food combinations and amounts to include in the diet, is relevant to support physical performance, weight management, and reduce disease risk. In terms of protein-containing foods, protein quality, and amount are two major considerations within the development of a healthy eating pattern irrespective of age. Food protein quality is traditionally dependent on its amino acid content and the availability of these amino acids in circulation, factors that would influence their metabolism within different body protein pools. Hence, protein quality is often based on protein digestibility ranking methods such as protein digestibility-corrected amino acid score (PDCAAS) or the digestible indispensable amino acid score (DIAAS), as will be highlighted below. The latter method has gained favor by the Food and Agriculture Organization of the United Nations (FAO) after the most recent review of the “best” methods to determine protein quality for human nutrition (1).

Regardless of the method used to measure protein digestibility in human foods (1), it is also important to consider coupling protein digestibility scoring metrics with other relevant human metabolic processes (2), such as the ability to influence protein turnover (i.e., synthesis and degradation) of body proteins. Given the primary role of dietary amino acids are to support protein metabolic demand and cover obligatory protein losses (3), it is perhaps important to consider coupling protein digestibility scoring methods with direct measurements of protein synthesis rates (e.g., within skeletal muscle) and whole body amino acid oxidation rates. For example, amino acids can only be “stored” within functional proteins, which given its size and nutrient sensitivity, positions skeletal muscle protein as a primary reservoir for dietary amino acids (4). Therefore, confirming that ingested protein foods are stimulating postprandial muscle protein synthesis rates without excessive amino acid oxidation rates provides confirmation that the available dietary amino acids in circulation are being used to support this vital tissue.

Protein requirements are set as a minimal need to prevent net nitrogen losses, but arguably are not sufficient to account for all factors contributing to quality of life throughout the lifespan (e.g., exercise habits, aging, hospitalization, or disease) (5). As such, there has been an impetus for a change for better a definition of “optimal” protein intake (5, 6). It has been suggested that greater focus on skeletal muscle is relevant when the goal is to define an optimal requirement of protein intake, especially throughout older adult life (7). The rationale behind this idea is that skeletal muscle represents a large proportion of total body protein in adults, or a large storage depot of energy and dietary amino acids, and contributes ~25–30% to whole body protein synthesis rates (8). Moreover, muscle has an obvious role in physical performance, but metabolically has important roles in the regulation of glucose disposal (*cf*. insulin resistance), fat oxidation, and energy balance (9). This ostensibly highlights its maintenance as especially pertinent throughout middle and old age. However, the increasing prevalence of metabolic disorders in pediatric populations and the potential for early programming of muscle tissue for later life (10, 11) likely refocuses the issue of optimizing muscle quantity and quality as being essential across the lifespan.

In this review, therefore, we discuss the role of dietary protein quality and quantity in terms of optimizing muscle mass from childhood to old age as a goal toward maintaining metabolic health and physical performance. We also discuss that a transition to a holistic framework within the area of protein nutrition is likely required to truly define optimal protein intakes for muscle. This involves shifting the focus from determining the effect of single nutrients (or the food parts) on metabolic outcomes, in favor of considering how an integrative holistic approach (e.g., exercise habits, eating pattern, and the food matrix in which the protein is consumed) affects the overall protein recommendation and associated muscle metabolic outcomes.

## Dietary Protein Quality

It is recommended that a healthy eating pattern consists of ingesting a variety of high quality protein foods to ensure a sufficient supply of amino acids for lean mass (e.g., muscle) maintenance or growth, and overall diet quality (12). In other words, the constituent amino acids of a protein food should match the requirement of the consumer and consist of a variety of protein foods to ensure nutrient density. Indeed, there has been a shift toward plant-based dietary patterns within dietary guidelines to presumably maximize population health benefits and to support environmental sustainability (13). For example, epidemiological findings suggest that minimizing red meat consumption within a dietary pattern is favorable to reduce disease risk (type 2 diabetes, cardiovascular disease, etc) (14, 15). However, these data are challenging to interpret as the comparison diet is often a confounding factor (e.g., different macronutrient compositions, processed vs. unprocessed, or varying fat percentages of meat intake) (16) and/or the lack of control of the physical activity patterns of the participants. It is also relevant to highlight that defining protein intakes based on very general definitions such as “ounce-equivalents” as provided by the USDA's Choose My Plate is discounting the value of protein quality scores and caloric intakes required to meet minimal essential amino acid requirements between animal vs. plant-based foods as indicated elsewhere (17).

The role of dietary protein quality is also perhaps relevant when defining more sustainable diets to meet the nutritional needs of an expanding global population (18). Protecting the planet (i.e., managing greenhouse gas emissions to land and water use) and living sustainably are also important topics in dietary protein quality considerations (19). As such, it is clear that there needs to be a range of methods to evaluate protein quality in order to titrate the claim for food as “high” quality depending on the desired physiological outcome. This also needs to be balanced against the potential environmental impact and importance of maximizing the use of natural resources for production of high quality proteins that provide target amounts of essential amino acids for muscle mass maintenance or growth (19).

DIAAS is the current protein quality ranking method that is recommended by the Food and Agriculture Organization of the United Nations (FAO) (20). The rationale behind this recommendation is that protein digestibility (quality) estimates should be based on true ileal digestibility (i.e., determined at the end of the small intestine where amino acids are absorbed), and ideally performed in humans. Thus, this method aims to determine what amino acid(s) may be limiting in circulation after accounting for digestion and absorption to support whole body protein metabolism. It is not feasible, however, to routinely perform ileal digestibility in humans. Hence, the growing pig model is often used due to the similarly between the digestive tract between pigs and humans, and the willingness of pigs to eat foods within the human diet (21). DIAAS cut-off values have been proposed to provide a basis for protein quality claims, while accounting for the quantity of protein ingested, such as excellent/high (100 or more), good (22–46), and no claim (<75) (20).

Much of the DIAAS work has been done on raw foodstuffs with more recent work focusing on how cooking method impacts food protein quality (47). This is relevant as many of the commonly consumed protein foods within the human diet have experienced heat treatment prior to consumption, which may impact its amino acid content and overall nutritional quality (48). As shown in Table 1, it has been established that cooking method (i.e., raw, boiled, grilled, pan-fried, or roasted) of meat affects its structural properties and subsequent DIAAS (47). It generally accepted that cooking (internal temperature of 70**°**C) increases protein digestibility by denaturing the protein and thus allowing greater bio-accessibility of proteolytic enzymes to its cleavage sites (47, 53). However, it was demonstrated that DIAAS was superior for the raw, boiled, and pan-fried minced beef conditions when compared to roasted or grilled beef in growing pigs (47). Collectively, these data highlight that the food matrix, such as food structure, can be manipulated by heat treatment to modulate protein quality scores. It is important to keep in mind, however, that severe heat treatment, or prolonged storage, can impact the nutritional value of amino acids (e.g., lysine) (54).

**Table 1:** Cooking method and its impact on protein quality scores.

	**Raw/Extruded**	**Boiled**	**Grilled/Baked**	**Pan-fried**	**Roasted**	**Source**
**Surface temp. (^**°**^C)**		****~**80**	****~**193–225**	****~**186**	****~**160**	
**Valine**						
Beef	0.97	0.99	0.80	0.98	0.91	(47)
Pinto beans	0.92	0.95	0.69			(49)
Green peas	0.93	0.98	0.89			(50)
Green lentils	0.80	0.93	0.86			(51)
**Isoleucine**						
Beef	1.25	1.25	1.11	1.23	1.15	(47)
Pinto beans	1.02	1.23	0.72			(49)
Green peas	1.03	1.16	1.06			(50)
Green lentils	1.11	1.05	0.91			(51)
**Leucine**						
Beef	1.09	1.11	0.97	1.08	0.99	(47)
Pinto beans	1.13	1.17	0.74			(49)
Green peas	1.00	1.13	1.00			(50)
Green lentils	1.02	1.04	0.83			(51)
**Lysine**						
Beef	1.28	1.21	1.11	1.11	1.12	(47)
Pinto beans	0.86	1.09	0.66			(49)
Green peas	1.07	1.15	1.10			(50)
Green lentils	1.05	1.04	0.79			(51)
**DIAAS**						
Beef	97^a^	99^a^	80^c^	98^a^	91^b^	(47)
Pinto beans	0.61	0.7	0.44			(49)
Green peas	0.7	0.67	0.7			(50)
Green lentils	0.53	0.49	0.44			(51)

*Beef internal temperature is 71 °C in all conditions. Within a row, values without a common superscript letter differ significantly (P < 0.001). Hodgkinson et al. (47) determined DIAAS directly based on true ileal amino acid digestibility using the growing pig model. Nosworthy et al. (49–51) estimated DIAAS based on fecal digestibility using a rat model. Lysine content does not represent reactive lysine and thus the bioavailability of digestible lysine might be overestimated (52). DIAAS, digestible indispensable amino acid scores*.

Certainly, it is more common to eat mixed meals, as opposed to single nutrients, throughout the day and thus it is relevant to have protein quality scores within the context of mixed foods/ingredients to better inform the various regulatory dietary frameworks (55). The challenge with this food-first approach could be identifying, let alone testing, the myriad of combinations of different food items to assess mixed food/ingredient interactions. However, research has begun to address this challenge through combinations of macronutrient co-ingestion. For example, in terms of protein digestibility, it has been established that the co-ingestion of lipids with protein improves protein digestibility/quality in growing pigs by slowing gastric emptying rates to allow more time for the ingested protein to be exposed to proteolytic enzymes and/or reducing passage rate within the small intestine to allow more time for the amino acids to be absorbed (56).

What is noteworthy, however, is that researchers have developed tools to assess the quality of dietary protein sources for the benefit of supporting whole body and muscle protein remodeling. Specifically, intrinsically labeled food proteins, whereby stable isotope tracers are incorporated into the protein matrix, are more readily applied within a human model to provide an index of protein digestibility and subsequent dietary amino acid availability after food ingestion (57–59). Using a labeled food protein approach, it has been established that macro-nutrient co-ingestion with isolated protein sources modulates postprandial protein derived amino acid availability in circulation, but not the stimulation of postprandial muscle proteins synthesis rates in healthy adults (60, 61). This highlights the potential disconnect between postprandial protein derived amino acid ability in circulation and the subsequent postprandial muscle protein synthetic response that may otherwise be missed without a metabolic tracer that can be tracked from mouth to muscle (60–63). These findings provide support for the notion that protein quality scores need to be coupled with other physiological correlates (e.g., protein turnover) to better define the impact of protein foods from a more “whole-human” perspective. This in turn will help inform healthy eating patterns and develop effective public health messaging toward the goal of optimizing muscle mass and health (2).

## Defining Optimal vs. Recommended Protein Intakes

Current protein recommendations, as defined by the recommended dietary allowance (RDA) or population reference intakes (PRI), throughout the lifespan are shown in Table 2. Protein recommendations are set as the lowest level of protein intake to prevent net nitrogen loss and reduce disease risk in nearly all (97–98%) healthy individuals at energy balance (64). However, these protein recommendation may not be optimal to support the metabolic needs of highly active individuals such as strength (65) and endurance trained populations (66). This is not completely surprising, however, given that protein requirements are designed to prevent protein deficiencies, which is particularly relevant for children and adults in developing countries but less of an issue in more developed nations (67). Therefore, lifestyle and goals of a given population (e.g., athletic performance, muscle growth/maintenance, functional independence, etc.) need to be considered when identifying minimum and optimal protein intakes.

**Table 2:** Protein recommendations throughout the life span as defined by the recommended dietary allowance (RDA), the population reference intakes (PRI), or muscle-centric meal-based recommendations.

**Protein recommendations**				
		**USA**	**Europe**	**Muscle-centric**
		**RDA (g/kg)**	**PRI (g/kg)**	**Meal-based (g/kg)**
**Across the lifespan^a^**				
Infants	(0–12 month)	1.50	1.31	?
Young children	(1–3 year)	1.10	1.01^b^	?
Children	(4–13 year)	0.95	0.90^b^	0.30^d^
Adolescents	(14–18 year)	0.85	0.86	0.30^d^
Adults	(19–70 year)	0.80	0.83	0.25
Pregnancy, lactation		1.10	1.07^c^	?
Aging Adult	(>70 years)	0.80	0.83	0.40

*Note that the RDA and PRI values are prescribed on a daily basis and obscuring the value of protein distribution and meal frequency as important factors for the stimulation of postprandial muscle protein synthesis rates. Meal-based recommendations should be consumed 4–5 times daily based on normally consumed meal-times (e.g., breakfast, lunch, snack, dinner, evening snack)*.

a Age ranges based on United States Department of Agriculture definition;

b mean of intake values for ages within given age range;

c calculated based on European Food Safety Authority absolute recommendation and reference female body weight;

d *based on whole body protein balance data. ? indicates unknown values*.

The “best” method to define an optimal protein intake is certainly a matter of debate (68–70), and will depend on the population studied (e.g., children or adults). Stable isotope tracer methods, such as the indicator amino acid oxidation (IAAO) or direct incorporation methods for the determination of muscle protein synthesis, have shown their utility to define protein recommendations across various ages and in relation to an exercise setting (71–74). We believe that studying nutrient requirements in the context of exercise should be a greater consideration as increasing levels of physical activity, including the incorporation of structured exercise regimes, is unquestionably one of the most important lifestyle behaviors for improved health (75), and is arguably our genetic “evolutionary default” as we were born to move. Importantly, exercise also directly affects nutrient utilization and requirement when compared to the sedentary-state. Hence, dietary and exercise guidelines are inherently connected and should be considered together when the goal is to define “optimal” protein intakes for improved health.

**Figure 1:**
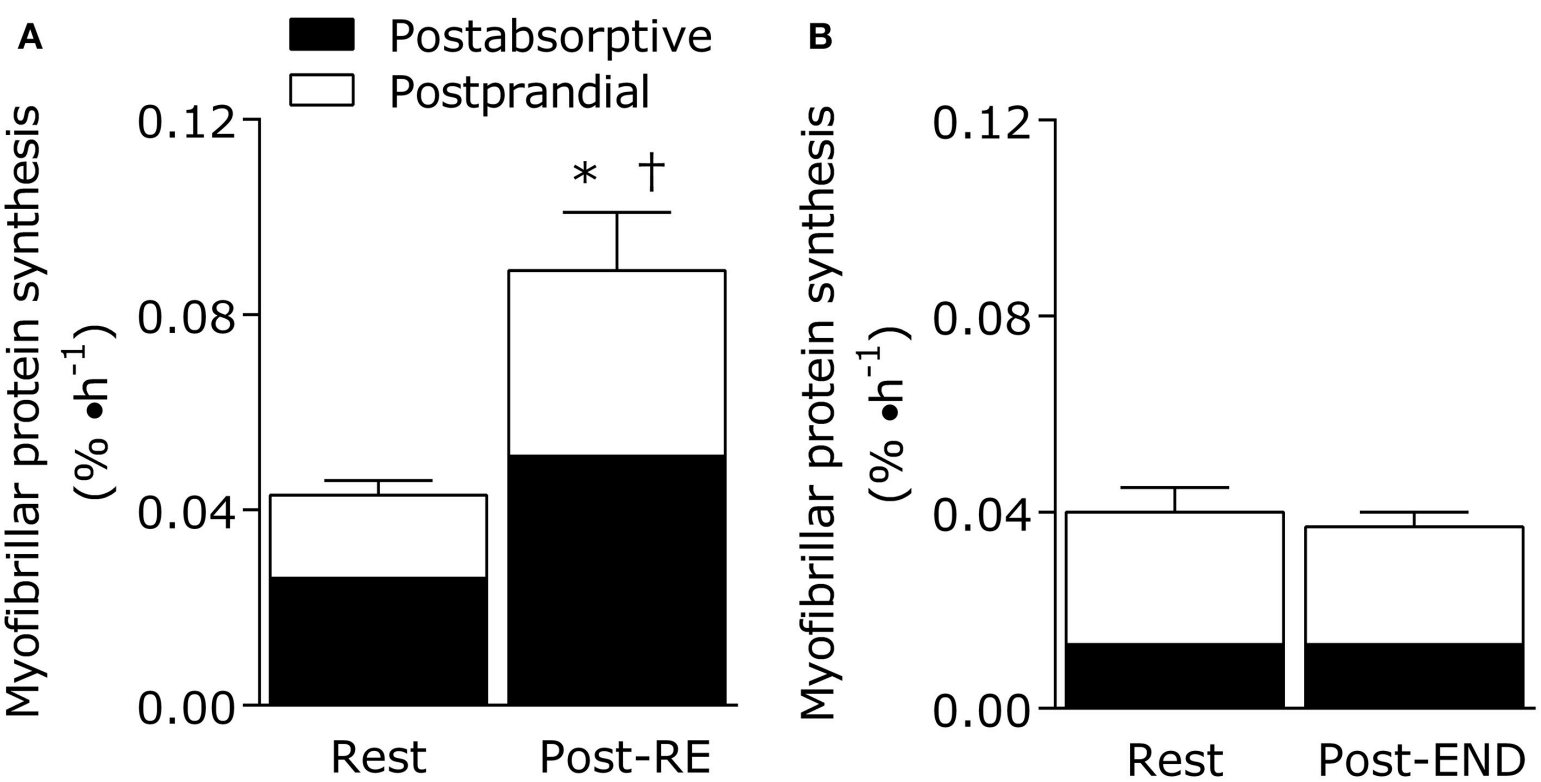
Eating an adequate amount of protein at rest (i.e., in absence of a prior exercise stimulus) generally results in a doubling of the myofibrillar (contractile) protein synthetic response from post-absorptive values in healthy young adults (20–35 years). The fundamentally anabolic nature of resistance exercise results in an interaction between feeding and the exercise stimulus during recovery such that the stimulation of postprandial myofibrillar protein synthesis rates is potentiated when compared to the resting value. This interaction on the stimulation of post-exercise myofibrillar protein synthesis rates is not observed during recovery from endurance exercise (treadmill running at 70% of VO2peak for 1 h). Data adapted from Burd et al. (76) and Abou Sawan et al. (77). *different from post-absorptive value at rest. †different from postprandial value at rest.

Importantly, exercise mode (strength vs. endurance exercise) directly impacts the metabolism of dietary protein at the whole body and muscle levels (Figure 1). For example, resistance exercise is inherently anabolic by improving net muscle protein balance (defined as muscle protein synthesis minus breakdown) for up to 2 days (78). Moreover, the performance of resistance exercise results in greater use of dietary amino acids for the stimulation of postprandial muscle protein synthesis rates during the immediate (0–4 h) (71, 79) and prolonged recovery period (~24 h) (76, 80). In other words, the ingestion of 10 g of essential amino acids (equivalent to ~25 g of high quality protein) is required to maximize the ingested protein dose-responsiveness of muscle protein synthesis rates in the sedentary-state (81). In the immediately post-exercise period, however, the ingestion of ~8.6 g of essential amino acids (equivalent to ~20 g of high quality protein) is required to plateau the postprandial muscle protein synthetic response (71). This implies that resistance exercise enhances the dietary amino acid sensitivity of muscle protein synthesis such that lower protein amounts are required to elicit a robust anabolic effect when compared to the sedentary-state. Similarly, it has been established that skeletal muscle tissue becomes a larger “sink” for dietary amino acids during recovery from resistance exercise as noted by the increased incorporation of dietary phenylalanine into muscle protein when compared to the sedentary-state (82). Finally, regular strength training results in increased whole-body nitrogen retention vs. the untrained-state (83). With these factors in mind, it seems that a greater ratio of circulating amino acids are being retained by the body's largest protein pool (skeletal muscle) in both the fasting and fed-states after resistance exercise. Such findings suggest that regular strength training is a strategy to optimize dietary protein utilization (Figure 1).

Interestingly, endurance exercise appears to be on the other end of the spectrum in terms of its impact on dietary protein utilization. Oxidation of endogenous amino acids may only represent a fraction of total energy provision during exercise (~2–10% depending on carbohydrate availability), but their utilization increases with endurance exercise intensity (84) and duration (85, 86). For example, estimates of leucine oxidation rates during moderate intensity exercise (~60% of maximal oxygen uptake; VO2max) are ~8 mg/(kg·h) (87) with rates increasing to ~10 mg/(kg·h) at higher intensities (~70% VO2max) (88) in endurance trained athletes. This may translate in a total leucine loss up to ~1.5 g over 2 h (89). Indeed, regular endurance exercise training blunts the exercise-induced stimulation of leucine oxidation rates (90), and it has been shown that 24 h net leucine balance remains unaffected by acute cycling exercise performed twice in a day (~50% VO2max for 90 min per session) (91). Thus, it could be speculated that there is a dietary protein accommodation occurring, thereby minimizing the extra demand on dietary protein with endurance exercise training (89).

However, our research groups have recently shown that 1 h of treadmill running at 70% VO2peak results in a stimulation of leucine oxidation rates and a net leucine balance that was more negative when compared to the resting-state in athletes (88). What is noteworthy is that net leucine balance remained negative throughout the postprandial period even when providing the athletes a generous amount of high quality protein (18 g whole egg protein) immediately after the acute bout (88). There was also no additive effect of nutrition and endurance exercise on the stimulation of post-exercise muscle protein synthesis rates in these athletes (Figure 1) (77), which is a hallmark of the muscle protein synthetic response during recovery from resistance exercise combined with feeding (71). These findings are significant (77, 88) as we provided an amount of protein (~0.25 g protein/kg per meal) immediately after the acute endurance bout that is commonly recommended to maximize the stimulation of post-exercise muscle protein synthesis rates after resistance exercise (71). Hence, we speculate that endurance exercise places more demand on dietary protein, which is likely intensity and exercise duration dependent, due to the need to compensate for exercise-induced amino acid oxidation losses while also supporting muscle protein remodeling throughout recovery when compared to resistance exercise. These concepts could be supported by recent estimations of an increased daily protein requirement (potentially primarily by the branched chain amino acids that are preferentially oxidized during exercise) to optimize whole body fed-state anabolism in endurance trained athletes during recovery (92, 93). Overall, protein recommendation for physically active adults are likely more nuanced whereby the “optimal” amount of protein to consume needs to take into account exercise mode, intensity, duration, and/or health/performance goals within the recommendation. This notion is consistent with periodized nutrition frameworks for carbohydrates commonly advocated to optimize training prescriptions and adaptations, especially for athletes (94).

Finally, it is also important to recognize that prescribing protein requirements as a single daily value as shown in Table 2 is likely obscuring the importance of protein distribution and meal frequency to optimize the postprandial muscle protein synthetic response throughout the day (95, 96). In short, dietary guidelines recognize healthy eating patterns for nutrient density and adequacy, but are currently not accounting for meal frequency. For example, it is common for adults, especially Americans, to skew their total protein intakes to dinner with smaller protein portions consumed at breakfast and lunch (96). Contrary to suggestions that there is not a practical maximal anabolic response to a meal protein intake (97), it is clear that muscle protein synthesis (71) and whole body net protein balance (73) have finite capacities to assimilate dietary amino acids. This would ultimately result in more dietary amino acids being irreversibly lost to oxidation as opposed to be used for postprandial muscle protein accretion at dinner when consuming a skewed daily protein distribution (71, 96). Thus, the definition of optimal protein intakes needs to consider meal frequency, and prescribe a recommendation on a meal-by-meal basis to take into account protein distribution as a relevant factor for the stimulation of postprandial muscle protein synthesis rates during the day.

## Protein Considerations for Children and Adolescents

The development of lean body mass during childhood and adolescence is important for supporting metabolic and skeletal health. Adherence to an active lifestyle is associated with greater lean body and muscle mass across the growth spectrum (98) and, due to the mechanical forces muscle may impose on growing bones, may be an independent predictor of peak bone mass (99, 100). Provided energy intakes are sufficient to support an active lifestyle and the metabolic demand for somatic growth, dietary protein represents arguably the most important macronutrient for the growth and development of lean mass.

General protein requirements are ~20–60% greater in children and adolescents than the minimum safe intake for adults to account for the metabolic demands of the linear and accelerated, respectively, growth of these young populations (101–103). Currently, the nitrogen balance-derived RDA is set at 0.95 g/kg/d and PRI at 0.90 g/kg/d, and is based largely on data from adult populations with an estimated growth requirement (determined by a factorial method) (102). In contrast, contemporary stable isotope-based methods (i.e., indicator amino acid oxidation) suggest the requirement to maximize whole body protein synthesis (as a metric for offsetting any fasted state protein loss) may be as high as 1.5 g/kg/d (103). However, with protein intakes at ~15% of energy, these recommended intakes are generally satisfied in the US when total energy intake is sufficient (22). Moreover, consideration for protein quality and individual amino acid requirements in children are unlikely to be an issue when consuming a typical mixed protein diet (i.e., plant and animal-based protein) at the current levels (23). It is important to note that regardless of method (i.e., nitrogen balance vs. IAAO), preliminary research suggests that, similar to adults, protein requirements in active children and adolescents may be (~50%) elevated, albeit relatively less than similarly active adults (10). This increased daily requirement may be related to a need to offset any exercise-induced losses and/or to support enhanced rates of lean body mass turnover and/or growth (10).

Dietary protein consumption in adults enhances the exercise-induced increase in whole body and skeletal muscle protein synthesis rates (4), the latter of which is generally the targeted outcome to aid in the remodeling and growth of this tissue in adults (24). In contrast to relatively weight stable adults, children experience whole body growth of ~5 cm height and ~3 kg body mass per year that may be accelerated 3-fold during the adolescent growth spurt (98). To accommodate for this somatic whole body growth that is enhanced via an active lifestyle (6), it is arguably more relevant to assess the nutritional factors that enhance whole body protein turnover and net protein balance (i.e., surrogate marker of acute “growth”) in children and adolescents. Similar to adults, protein consumption after exercise increases whole body net protein balance in children and adolescents in a dose-dependent fashion (25, 26, 73). Perhaps consistent with the requirement to support whole body growth, active children, and adolescents appear to be more “anabolically sensitive” to dietary protein than adults as whole body net protein balance is greater in these young populations at suboptimal (i.e., < ~0.3 g/kg) meal protein intakes (10). However, similar to adults, whole body net protein balance is saturable with protein ingestion in active children and adolescents (26, 73). For example, whole body leucine oxidation rates (estimate of protein oxidation) plateaus at an intake of ~34 mg leucine/kg (equivalent of ~0.34 g/kg of a high quality, leucine-enriched protein) with greater intakes resulting in an expansion of plasma amino acid pool (26), which represents a metabolic profile that could be suggestive of an acute nutrient excess (27). Therefore, available data suggests children and adolescents should target a meal protein intake of ~0.3 g/kg to maximize whole body net protein balance during the recovery from acute exercise (26, 73), an intake that incidentally has also been shown to maximize post-exercise muscle protein synthesis in adults (71).

The timing and distribution of protein intake throughout the day has been suggested to represent a modifiable factor to optimize dietary protein utilization in adults (95). Similar to adults, children in the United States have been reported to consume a skewed protein distribution with the majority of the daily intake consumed in the evening (28). Whereas, there is some support for consuming a balanced daily protein distribution to enhance protein balance in children (29, 30), this finding is not universal (31). It is possible that the nutrient demands for growth in active children and adolescents render them more sensitive to dietary amino acids and, thus, less influenced by variations in protein distribution. This may be akin to the ability of resistance exercise in adults, the arguable only parallel to “growth” in this population, to increase the sensitivity of muscle protein synthesis to dietary amino acids for up to 24 h (76). Nevertheless, given the anabolic response to bolus protein ingestion is saturable, prudent advice may be to target the repeated ingestion of moderate protein-containing meals to optimize the anabolic efficiency of the daily protein intake. Similar to adults, however, additional research is warranted to identify the anabolic potential of different protein sources independently and within whole food matrices and mixed meals.

## Protein Considerations With Age

It is well-established that there is a gradual loss of skeletal muscle mass and function that occurs at a more advanced age, and that this muscle deconditioning is usually coupled to sedentary lifestyle behaviors (32). For example, the age-related loss of skeletal muscle mass is thought to begin at ~50 years and progress at a rate of ~0.8% per year (33) whereas the decline in strength, while associated with muscle loss, occurs at a faster rate of ~2–3% per year (34). Therefore, when an individual reaches 70 years of age, they may have lost ~16% of their muscle mass and ~50% of their strength from their younger years.

The age-related decline in overall skeletal muscle mass can be attributed to an imbalance between muscle protein synthesis and breakdown rates that results in a negative muscle protein balance (35). No detectable differences shown to exist in post-absorptive muscle protein synthetic rates between younger and older men (36, 81) and women (37). Hence, the age-related decline in muscle mass is thought be attributed to the blunting of the postprandial muscle protein synthetic response to protein ingestion when compared to their younger counter-parts (36, 38, 81). The impaired ability of aging muscle to elicit a robust postprandial muscle protein synthetic response to elevated dietary amino acid availability in circulation has been coined “anabolic resistance” (39). Various strategies have been used in an attempt to overcome this age-related anabolic resistance of muscle protein synthesis rate such as increasing the protein density of meals (40, 41), food fortification techniques including extra leucine as an anabolic trigger (42), and food combinations (60, 61). However, what appears to be the most promising, and cost effective, lifestyle strategy to improve the postprandial muscle protein synthetic response to protein ingestion at a more advanced age is regular exercise (82). The final point that has received little attention is the potential sexual dimorphism in the age-related changes in muscle protein synthesis rates in response to protein. There is some indication that aging men and women may respond differently to nutritional stimuli (43, 44), but both sexes are clearly anabolically resistant (43). At this time, however, there is not enough data to clearly define if older women have different protein requirements when compared to older men.

Despite this established anabolic resistance with age, current protein requirements as established by whole body nitrogen balance methods are similar throughout adult life (Table 2). When using a muscle-centric approach to protein intake, however, we have observed that the relative quantity of protein to maximize the postprandial muscle protein synthetic response is greater in older when compared to younger men. In particular, we established that older men demonstrated an ingested protein-dose response curve of postprandial muscle protein synthesis rates up to ~0.40 g/kg per meal, which was nearly doubled when compared to young adults (~0.24 g/kg per meal) (72). When considering the value of spread distribution pattern of protein intake at each meal time (i.e., breakfast, lunch, dinner, and evening snack) for maximal muscle anabolic potential (45, 96), it seems that protein intakes for older adults is likely higher than the current RDA or PRI of ~0.8 g/kg/d and nearing values closer to ≥1.2 g/kg/d. These recommendations are supported by whole body tracer estimates using the indicator amino acid oxidation technique of a safe intake of ~1.25 g/kg/d in older (i.e., >65 years) adults (46). In addition, lean body mass loss over 3 years is lowest in older adults consuming ≥1.2 g/kg/d (104), collectively supporting dietary protein as a modifiable risk factor for age-related lean (and muscle) loss. However, a prospective multi-site randomized control trial with defined protein intakes spanning sufficient to deficient with consideration for habitual activity and functional endpoints (e.g., muscle strength/mass) is ultimately needed to guide best practices in nutritional advice.

## Holistic Approach for Better Definitions of Optimal Protein Intake for Muscle?

Reductionist approaches have made significant contributions toward the understanding of nutrient-muscle interactions. For example, it has been established that dietary protein derived amino acids, especially the essential amino acids (105), are mainly responsible for the stimulation of postprandial muscle protein synthesis rates. Moreover, the branched chain amino acid, leucine, has received much attention due to its dual role as an anabolic signaling molecule (106, 107) as well as a substrate for protein synthesis (108, 109). However, with the general preoccupation of the field studying the individual parts (i.e., isolated proteins and free amino acids) of nutrition in a typical bottom-up fashion, our current approach to understanding human nutrition may be nearing its limits to adequately define the role of protein quality and quantity for muscle mass and health within a complete diet.

**Figure 2:**
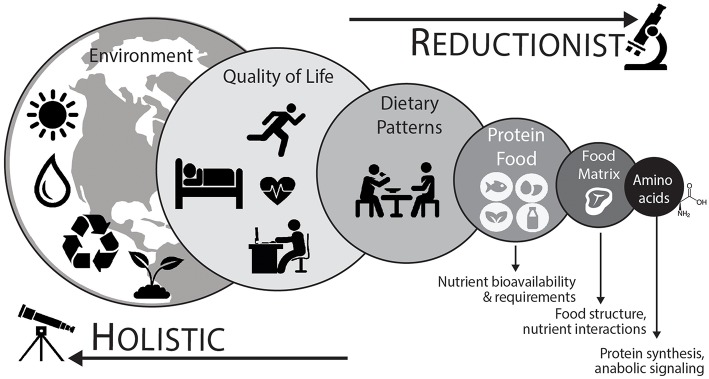
To adequately define optimal protein intakes it is important to consider an integrative holistic approach. This “top-down” approach considers that different levels are additive to the next for the development of dietary advice (110, 111). Dietary patterns (animal based vs. plant based) and their associated protein foods are directly connected. Protein food is more than the sum of its constituent amino acids and the net effect of the food matrix, or food combinations (e.g., complementary protein pairing of plant-based foods), likely has an impact on the stimulation of postprandial muscle protein synthetic responses and overall diet quality. At the highest levels, food sustainability, food waste, and other human choices are important considerations. At the lowest (reductionist) level, amino acids represent the fundamental building blocks of protein and are anabolic agents in themselves (i.e., initiate protein synthesis). Aside from nutrient factors, ample physical activity, including regular structured exercise, is important component of a healthy lifestyle and has a direct impact on protein utilization and the overall nutritional recommendation.

As shown in Figure 2, a holistic point-of-view considers that protein nutrition follows a hierarchical organization with each level demonstrating a reinforcing factor into the next for the overall protein recommendation (110, 112). Using a top-down approach, which takes into account environmental (e.g., time of year, geographical location, and sustainable agricultural practices), quality of life (e.g., physical activity/exercise habits or injury), dietary pattern (e.g., Western, Mediterranean, or vegetarian), protein foods (e.g., beef or quinoa), net effect of the food matrix (e.g., food structure and nutrient-nutrient interactions), and finally the most basic constituent of protein (i.e., dietary amino acids), will help advance the field of research and perhaps yield the most ecologically valid dietary advice (112, 113).

At the higher levels, it is important to first consider the eating pattern of a population as dietary guidelines consist of eating patterns and their respective food choices to ensure nutrient adequacy and overall diet quality. Dietary eating patterns are often adapted to meet personal preference with common patterns including animal based (e.g., US-style) or plant-based (e.g., vegetarian) eating patterns. Indeed, plant-based diets are often thought to be inferior for the stimulation of postprandial muscle protein synthesis (114). Plant based foods, when viewed in isolation, are lower in leucine, lysine, and methionine by total amino acid content when compared to animal based foods (115). As such, it has been demonstrated that the ingestion of soy protein isolate resulted in a reduced postprandial muscle protein synthetic response when compared to whey protein ingestion in healthy young men (116). However, vegetarian and vegan diets are quite diverse, and generally consist of the ingestion of a variety of plant based foods throughout the day to ensure a more balanced profile of essential amino acids for the stimulation of postprandial muscle protein synthesis rates (117). Direct comparisons, however, are non-existent with regards to the capacity of mixed plant based foods to augment postprandial muscle protein synthesis rates vs. the ingestion of animal based foods.

It is also significant to develop protein recommendations in relation to whole food approaches, which takes into account the amino acid composition of the ingested protein food as well as the associated net effect of the food matrix (118). The food matrix describes the nutrient and non-nutrient components of foods as well as their structure and interactions (113, 119). The food matrix can influence nutrient digestion, absorption, and in terms of protein containing food matrices, the net anabolic action on the stimulation of muscle protein synthesis rates (62, 120–123). Such findings strongly suggest that there are interactions occurring within the food matrix to potentiate the net muscle anabolic effect that is stronger than the individual action of amino acids alone (118). Overall, dietary patterns are composed of foods, food combinations, and their associated food components and nutrients. Certainly, it is relevant to deconstruct dietary patterns, and subsequently understand how the parts of foods (i.e., amino acids) activate anabolic signaling pathways and stimulate the postprandial muscle protein synthetic response to understand the mechanistic basis behind a dietary recommendation. However, it is also important to balance the knowledge gained from studying isolated food components with the interactions occurring between exercise habits, eating patterns, and foods (and their constituent nutrients) when providing dietary advice (Figure 2).

## Conclusion

Identifying the optimal amount and quality of protein foods to consume within a dietary pattern is necessary to provide dietary guidance. We have discussed optimal protein intakes from a muscle-centric point of view given its role in muscle function and metabolic health. There is little uncertainty that there needs to be some level of flexibility when considering what is the “optimal” protein intake to include within a dietary pattern throughout the lifespan. In terms of the protein RDA or PRI, these values represent a minimal target to prevent a protein deficiency within a safety margin, and perhaps are not adequate to support muscle protein remodeling with regular exercise training (6) and/or account for the increased dietary protein amounts required to overcome anabolically resistant aged muscles (7). Moreover, protein quality is also an important consideration of a dietary plan. The DIAAS of a dietary protein may yield more direct information with regards to protein digestibility (2), but there is currently limited DIAAS available based on a wide variety of dietary proteins. Moreover, DIAAS does not consider the impact of exercise training on modulating protein digestibility and the transfer of bioactive food constitutes (118), which will play a role in defining optimal protein quality.

At some point, it is also important to recognize a holistic nutrition framework where there is interplay between environmental considerations, physical activity and exercise patterns, dietary patterns, protein foods, and nutrients (amino acids) that cultivates into the overall dietary advice (Figure 2). Likewise, it is essential to keep in mind that there is adaptability for any protein recommendation throughout the life/health-stage, which accounts for health or performance goals, periods of hospitalization, or disease-state. In turn, this will provide a better compass for the definition of “optimal” protein intakes for all ages.

## Author Contributions

NB, KP, and DM drafted the manuscript. KP, AS, and CM prepared tables and figures. All authors contributed to manuscript revision, read, and approved the submitted version.

### Conflict of Interest Statement

NB has received research grants, consulting fees, and speaking honoraria from PepsiCo, the National Cattlemen's Beef Association, and Alliance for Potato Research and Education (APRE). DM has received research grants, consulting fees, and speaking honoraria from Nestec S.A., Ajinomoto Co. Inc., Dairy Management Incorporated, and Iovate Health Sciences International. The remaining authors declare that the research was conducted in the absence of any commercial or financial relationships that could be construed as a potential conflict of interest.
